# Diagnostic and Therapeutic Challenges of Catatonia in an Adolescent With High Functioning Autism Spectrum Disorder: A Case Report

**DOI:** 10.3389/fpsyt.2021.644727

**Published:** 2021-06-02

**Authors:** Annalisa Traverso, Caterina Ancora, Silvia Zanato, Alessia Raffagnato, Michela Gatta

**Affiliations:** Child and Adolescent Neuropsychiatry Unit, Department of Women's and Children's Health, Padua University Hospital, Padua, Italy

**Keywords:** catatonia, autism spectrum disorder, high functioning autism, ECT, adolescent psychiatry

## Abstract

Catatonia is a psychomotor syndrome with specific clusters of speech, behavioral and motor features. Although potentially life-threatening, especially in its malignant form accompanied with autonomic dysregulation and medical complications, it is a treatable condition, when promptly identified. For a long time catatonia was considered a marker of schizophrenia, thus limiting the possibility of diagnosis and treatment. Due to growing awareness and studies on the subject, it is now known that catatonia can occur in the context of a number of diseases, including psychotic, affective and neurodevelopmental disorders. In recent years, there's been a renewed interest in the recognition and definition of catatonia in neurodevelopmental disorders, such as Autism Spectrum Disorder (ASD), where the differential diagnosis poses great challenges, given the considerable overlapping of signs and symptoms between the conditions. We present the case of a 15 year old boy with High Functioning ASD with a sudden onset of severe catatonic symptoms and the co-existence of psychotic symptoms, whose complex clinical course raises many questions on the differentiation and relation of said disorders.

## Introduction

### Catatonia and Autism Spectrum Disorder

Catatonia is a psychomotor syndrome with specific clusters of speech, behavioral, and motor features. Although potentially life-threatening, especially in its malignant form accompanied with autonomic dysregulation and medical complications, it is a treatable condition, when promptly identified. Symptoms include stupor, catalepsy, waxy flexibility, posturing, mutism, negativism, stereotypic movements, mannerisms, echo-phenomena, grimacing, agitation (1), however more than 40 manifestations have been described in the literature (2).

After being tied to schizophrenia for more than a century, thanks to growing interest and studies on the subject, it is now known that catatonia can occur in the context of a number of diseases, including psychotic, affective and neurodevelopmental disorders, and of other medical conditions (e.g., metabolic, endocrine, rheumatologic, neurologic, autoimmune, and paraneoplastic disorders), substance abuse or withdrawal (1, 3–6).

In the fifth edition of the Diagnostic and Statistical Manual of Mental Disorders (DSM-5), catatonia began to be recognized as a self-standing entity, with the category “unspecified catatonia,” in addition to “catatonia associated with another mental disorder” and “catatonic disorder due to another medical condition” (1). According to the DSM-5, catatonia can be diagnosed with the presence of three or more of 12 psychomotor core symptoms, ranging from unresponsiveness and decreased motor activity to agitation and peculiar motor activity, among the others. The variety of seemingly opposing clinical features could be responsible for the relative lack of awareness and recognition of the condition among the scientific community through the years.

This is particularly significant, since catatonia is a treatable condition, responding to benzodiazepines and electroconvulsive therapy (2, 7–11).

Often underdiagnosed, to the point of being considered “hidden in plain sight” among other disorders, catatonia in the pediatric and adolescent population is not so rare (12). The prevalence of catatonia among pediatric psychiatric inpatients was estimated between 0.6 and 17 % (13–17). Despite being a treatable condition, with a specific, symptomatic approach, which proved to be efficient among the pediatric and adult population (18), it is important to note that catatonia carries one of the highest risks of mortality in the psychiatric setting (19).

Many conditions (Phelan McDermid syndrome, Kleine-Levin syndrome, Prader-Willi syndrome, 22q11.2 deletion syndrome, autoimmune encephalitis) show common elements with catatonia, regarding their etiology and pathophysiology (12, 20) and a thorough assessment needs to be performed whenever the suspicion of catatonic symptoms is raised.

In recent years, there's been a renewed interest in the recognition and definition of catatonia, specifically regarding neurodevelopmental disorders, such as Autism Spectrum Disorder (ASD), where the differential diagnosis poses great challenges, given the considerable overlapping of signs and symptoms between the conditions (20).

Autism Spectrum Disorder (ASD) is a pervasive neurodevelopmental disorder characterized by deficits in social interaction, communication, and restrictive and repetitive behaviors, usually diagnosed during childhood (21–23).

In the 2010 Global Burden of Disease study, worldwide autism prevalence was estimated 1 in 132 individuals (24, 25), but numbers are much higher in high-income countries, such as the United States, where the estimated prevalence in 2016 was 18.5 per 1,000 (one in 54) children aged 8 years, being 4.3 times more prevalent among boys than girls (26).

The term High Functioning Autism, which is not contained in the DSM, is commonly used by clinicians to identify ASD patients with average intellectual abilities (IQ of 70 or greater).

High rates of catatonic symptoms have been reported in ASD patients, to the point of being recognized as a possible associated feature of ASD in the DSM-5. Wing and Shah, in a systematic examination of 506 individuals with ASD, compared with controls, found that 17% presented catatonic features (27). Most of them were males, with symptoms onset in the age range of 10–19 years and intellectual disability was a risk factor.

There are a multitude of hypothesis regarding the pathophysiology of catatonia, including neurotransmitter, genetic, metabolic abnormalities and psycho-sociological factors such as trauma and severe stress (20, 28), but the etiology in most cases remains unclear.

We present the case of a 15 year old boy with High Functioning ASD (HF ASD) with a sudden onset of severe catatonic symptoms and the co-existence of psychotic symptoms, whose complex clinical course raises many questions on the differentiation and relation of said disorders.

## Case Report

P is a 15 year old boy in good physical health. He has no siblings and lives with his parents in the countryside. His family has no history of mental disorders.

He was born without complications after a full-term pregnancy.

He was diagnosed with Autism Spectrum Disorder at 3 years as he presented mild developmental delay, persistent deficits in social communication and repetitive patterns of interests. Full assessment included the ADOS (Autism Diagnostic Observation Schedule) (29), the ADI-R (Autism Diagnostic Interview–Revised), a cognitive evaluation and the Vineland adaptive behavior scale. Genetic testing (array-based Comparative Genomic Hybridization, FMR1, and MECP2 analysis) was negative.

P received psychomotor and speech therapy, at-home educational support and social skills training. He was able to achieve a good development of adaptive and daily-living skills. Throughout the years, P attended school with a support teacher, without requiring a special curricular program. His academic achievements were adequate. His cognitive evaluation (Wechsler Intelligence Scale for Children-IV) at 13 showed a full scale IQ score of 107, with a major discrepancy between the Verbal Comprehension Index (VCI = 84) and the Visual Spatial Index (VSI = 130) (30). He was fluent in two languages, traveled extensively with his family and practiced sports at competitive levels. He developed interests in cinema, Japanese culture and music. He had a small number of close friends and wished to become a film-maker.

At 15 years of age, while studying, P manifested brief episodes, lasting about 3 to 5 min, of apparent stupor. After a few days, he presented a longer episode of abrupt arrest of ongoing activity and unresponsiveness to verbal stimuli. He was taken to the Emergency Room, where he seemed to experience an acute onset of visual hallucinations (seeing a red dragon), along with psychomotor agitation.

He was hospitalized the following day, as he presented with severe symptoms of catatonia (stupor, mutism, posturing, psychological pillow) with rigidity, failure to swallow and urine retention.

He was treated with a challenge dose of 2 mg of intravenous lorazepam with immediate response.

This improvement, though, appeared fluctuating. In his first days as an inpatient, P manifested episodes of regurgitation of gastric contents and autonomic instability with daily variations of body temperature (up to 37.4°C) and of heart rate (up to 130 bpm), skin flushing, diaphoresis. His Bush Francis Catatonia Rating Score (BFCRS) was (31). P also presented a wide array of symptoms, including disorganized thought and uninhibited, sometimes sexualized, behavior. Episodes of agitation, where he appeared to have visual hallucinations, persisted. Therefore, treatment with low doses of haloperidol was started and subsequently switched to aripiprazole, given its more favorable pharmacologic profile with lower risk of extrapyramidal side effects (32).

An extensive pediatric neurological and metabolic assessment was inconclusive. Wake and sleep electroencephalograms, brain MRI, CSF analysis, immunology (including anti-NMDAR, AQP4, GABA-B-R, AMPAR, VGKC antibodies) and microbiology were normal, however a slight blood-brain barrier damage was documented, but deemed unspecific. Metabolic tests on CSF (dosing of amino acids, pterins, and neurotransmitters) showed decreased 5-hydroxyindoleacetic acid (5-HIAA) and homovanillic acid. This finding was considered unremarkable, probably secondary to neuroleptic medications.

During the first month of hospitalization, P seemed to fluctuate between mutism with immobility and rigidity, and bursts of disinhibition and hyperactivity, sometimes showing aggressive or bizarre behavior. In most of his mental state examinations P appeared alert, although scarcely compliant, bradykinetic and bradylalic. He could execute simple commands inconsistently. His gaze appeared blank, although he would blink to threat. Grimaces and stereotypies (such as body rocking) were at times prominent. His insight seemed partial.

His response to lorazepam was incomplete. He presented episodes where he would freeze during movements and get stuck halfway through motor actions, maintaining odd postures against gravity, in a waxing and waning fashion. P also showed obsessive-compulsive symptoms (such as counting backwards from big numbers) and obsessive slowness. Due to the onset of gross and fine tremor and worsening of excessive salivation, an anticholinergic medication (biperiden hydrochloride) was added.

As an inpatient, P was provided with psychological support. Engagement in psychotherapy was initially impossible, for he would show minimal interaction and a pervasive focus on sensorial stimuli. As he started to open up more, P revealed that he had been the subject of bullying (physical and verbal aggression) by some of his classmates.

After P reported self-harming and potentially suicidal intentions, treatment with lithium carbonate was initiated.

His drug regimen consisted of lorazepam 8 mg/day (the administration was gradually switched from iv to PO), aripiprazole 20 mg, lithium up to 900 mg/day. Due to low tolerability, lithium and aripiprazole were gradually reduced.

Gradually, P showed a slight improvement in motor functions, feeding and swallowing. Daily changes in his mood and appearance became noticeable: in the morning he seemed sedated, while in the afternoon he was awake and alert, talked with other patients, walked along the corridors, played card games.

P was discharged after 3 months as an inpatient, with a diagnosis of severe catatonic episode and affective decompensation with psychotic symptoms. He showed general improvement, although maintaining important behavioral fluctuations throughout the day. P had lost 5 kg from admission and was borderline underweight.

Pharmacological treatment at discharge consisted of lithium 450 mg/day, aripiprazole 10 mg/day, lorazepam 0.5 mg/day, biperiden hydrochloride 2 mg/day. His BFCRS was 17. An Intelligence Test (WISC-IV) was performed. It showed cognitive deterioration with IQ score 82, having lost more than 20 points in Visual Spatial Index, Working Memory and Processing Speed Index, compared with the previous testing of 2016, administered by the same Specialist.

P started a rehabilitation program with Applied Behavioral Analysis (ABA) therapy, integrated with physical prompting. Regular follow-up evaluations were conducted. Although his family was greatly involved in the therapeutic process, they seemed to be losing trust in medications, and asked for them to be further tapered down.

For 1 to 2 months, during the summer, P appeared to be relatively well, with moderate functional independence.

A few months later, mood changes and obsessive-compulsive symptoms became evident. Treatment with fluoxetine (up to 20 mg/day) was started. At this time, P needed physical prompts to initiate and complete almost every movement and required assistance for his daily living activities, at home and at school. Facilitated communication techniques were applied, due to increasingly persistent mutism.

Gradually, P started to communicate more through writing and typing. At times, he exhibited brief windows of wellness, where he seemed to be back to his premorbid self. Overall he showed major daily fluctuations in his behavior, especially with symptoms of negativism toward his parents and caregivers.

P continued to undergo psychoeducational therapy with a specialized team made of psychologists and occupational therapists, working on a program tailored to his needs, while attending regular psychiatric and pediatric checks. Brain MRI and electroencephalogram were repeated and both turned out normal.

After 1 year, P's condition has substantially improved, but his functioning has not returned to baseline.

**Figure 1:**
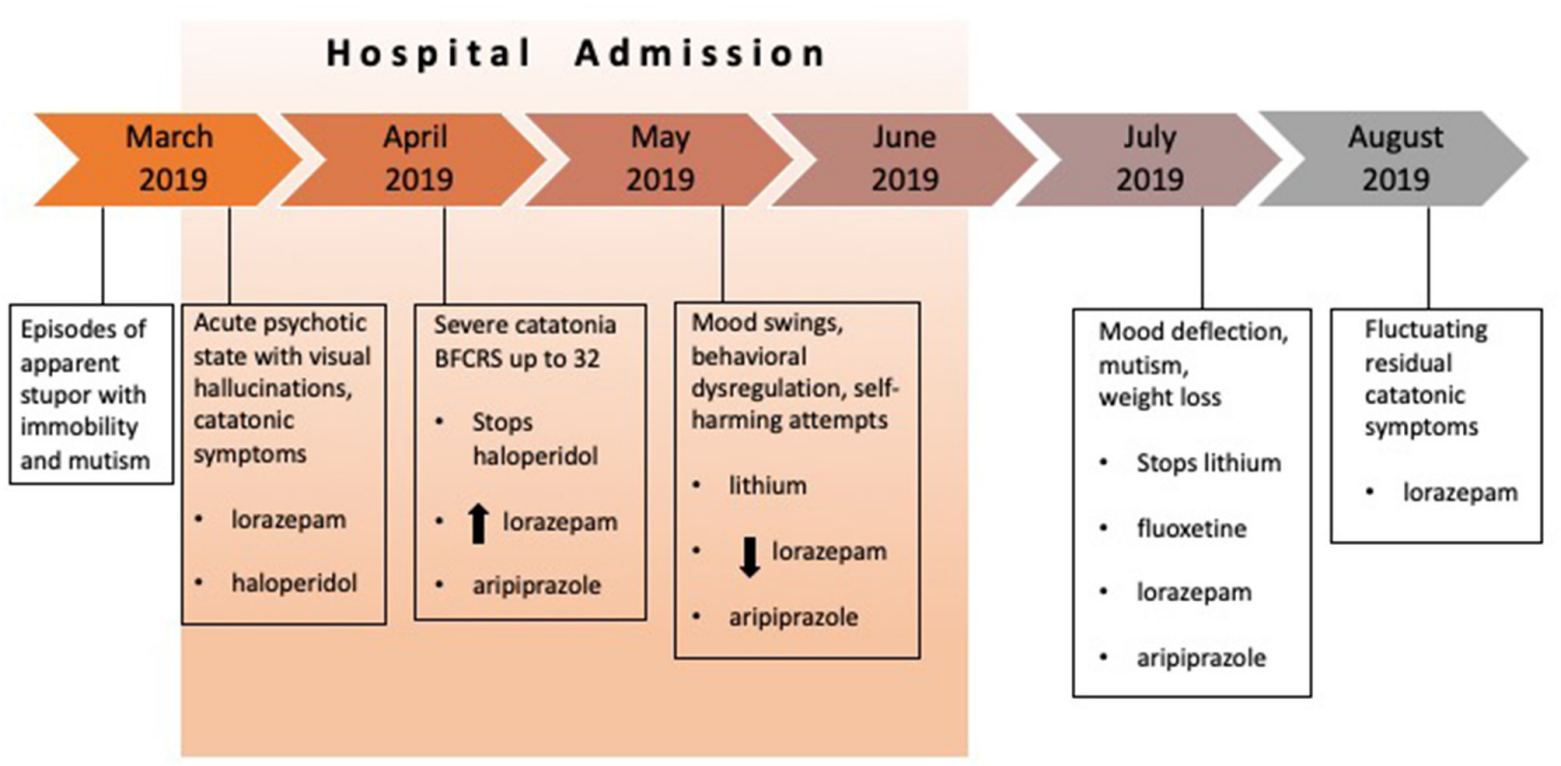
Patient's clinical course and treatment.

Fluoxetine, neuroleptics and lithium were stopped due to lack of consistent efficacy and by family's choice. A small dose of lorazepam was maintained (1 mg/day), with adequate adherence and tolerability. His parents reported their unwillingness in accepting further pharmacological interventions See Figure 1. When asked, P seemed ambivalent about the possibility of receiving further treatment.

## Discussion

P's case is emblematic of the myriad challenges of diagnostic overshadowing of neuropsychiatric disorders.

The acute onset of P's symptoms, their range and variability throughout the day in a waxing and waning course and the incomplete response to benzodiazepines made it arduous to identify P's condition under a single diagnostic framework. This, along with the susceptibility to pharmacological adverse effects, contributed to the difficulties with parents' compliance to treatment.

P presented with catatonic and psychotic symptoms and received medication for both. The treatment with lorazepam in adequate doses produced an improvement of P's symptoms, but not a complete remission. It's important to note that patients with catatonia and ASD tend to respond less consistently to benzodiazepines than those with mood disorders (33). It could be hypothesized that, among ASD patients, those with a higher functioning might be at greater risk of receiving multiple medications (such as antipsychotics and antidepressants), as they could be more capable of reporting their internal experiences.

Psychomotor disturbances, social withdrawal, mutism, stereotypy, echo phenomena are characteristics of both Schizophrenia Spectrum Disorders (SSD) and ASD (34). The presence of hallucinations in ASD, although not common, has been reported, especially in High Functioning ASD (35, 36). It might be argued that the use of a first generation antipsychotic such as haloperidol, even if in low doses, contained hallucinations and agitation, but worsened catatonic features. According to many authors, Neuroleptic Malignant Syndrome (NMS) can be considered as a subtype of catatonia, consequent to the use of neuroleptic medications (37, 38) and they both require treatment with GABAergic drugs, Electroconvulsive therapy (ECT) and supportive care (39–41). Given the onset of mild autonomic instability, it could be inferred that P suffered from a case of catatonia with malignant traits. Malignant catatonia, previously named “lethal catatonia” is considered the most severe form within the spectrum of catatonic syndromes, with potential risks of serious complications, including pneumonia, decubitus ulcers, thrombosis, malnutrition, dehydration, rhabdomyolysis, and consistent mortality rates (42).

The onset of obsessive-compulsive symptoms and rituals could further complicate the diagnostic process, bringing in the picture of the most severe type of Obsessive-Compulsive Disorder (OCD), Obsessional Slowness (43).

The prevalence of psychiatric comorbidity among ASD patients is estimated to be around 70%, and among the most frequent diagnoses are mood disorders, anxiety and obsessive compulsive disorder (OCD) (44–46).

The rate of anxiety disorder in ASD youth was calculated to be ~40% (47). In this population, it can be difficult to identify the symptoms of anxiety, for they can be misinterpreted as ASD symptoms or ASD-related difficulties (48). The same risk applies to mood disorders (Major Depressive Disorder and Bipolar Disorder), as the symptoms in ASD patients might be atypical, ranging from worsening of core ASD traits (stereotypies, selective interests) to regression of previously acquired skills and self-injury behaviors (49–52).

Although no consistent benefit is documented in response to antidepressant medications and mood stabilizers, these agents are often administered in clinical practice, as were in this case.

The appearance of catatonic patients, with stone-like rigidity and often terrified facial expressions, has led researchers to link catatonia with extreme fear, interpreting it as an evolutionary, defensive, response to fatal danger (53). The implications of trauma and severe stress, such as being a victim of bullying, could also bring into consideration PTSD (Post-Traumatic Stress Disorder) and the recently recognized Resignation Syndrome, seen in psychologically traumatized children in the process of seeking asylum in Sweden (54). The prevalence of PTSD among ASD patients is difficult to establish and its characterization is in need of extensive studies (55). It is important to note that, in the general population, victims of bullying have consistently higher rates of anxiety, depression and suicidality, compared with controls of the same age (56–58). ASD children and adolescents are considered in many studies a particularly vulnerable population, with prevalence as being victims ranging from 7 to 75% (59–61), to the point of bullying being one of the main daily stressors of ASD youth (62).

The characteristics of presentation and severity of symptoms, in P's case and in other cases in literature, seem to be waxing and waning, within a general picture of chronic deterioration of functions. Historically, alternation of stupor and agitation was introduced by Kraepelin as a core symptom of catatonia (63). Later, Leonhard recognized a specific type of catatonia, called Periodic Catatonia (64), characterized by a temporal fluctuation of manifestations. In our experience, managing a patient with such diverse symptoms, recurring with a rapidly changing pattern, proved to be most challenging, especially regarding the choice and duration of drug treatment. During P's phases of symptoms remission medications were often tapered down, only to be increased again during episodes of stupor or intense agitation. This periodic course of symptoms is characteristic of catatonia and should not lead to premature changes in treatment, especially in situations where a disorder's core signs might be masked by ASD features.

Catatonia, psychosis and ASD have long been considered competing diagnoses or, more recently, entities belonging to a wide spectrum, flip sides of the same coin, apexes of an iron triangle (34, 65, 66). The metaphors are countless, and explicative of the diagnostic and therapeutic complexity of these diseases.

This case proves to be a good example of the many challenges of diagnostic overlapping in the field of Pediatric Neuropsychiatry and, more precisely, in that of Autism Spectrum Disorders.

If promptly recognized, catatonia is a treatable condition, responding in a majority of cases to benzodiazepines. The second line therapy for benzodiazepine-resistant catatonia is ECT, but the practice in many countries, including Italy, is strictly regulated and restricted, with very few centers offering it, making it difficult to apply, especially in the pediatric population. Catatonia, particularly in its malignant form, is considered an indication for ECT, with high rates of remission with maintenance therapy (67). In these instances, ECT is a life-saving procedure, and its application should be considered early. In recent years, a growing number of reports and studies have recognized the efficacy of ECT in the treatment of catatonia among the pediatric population, including ASD patients (68–71), with favorable response rates (39, 66) indicating that ECT can be a definitive treatment of catatonia in these cases (67, 72). Although ECT was first introduced in Italy by Cerletti and Bini in 1938, its use in Italy is now dramatically averted, due to concerns about safety and ethical issues. Controlled clinical trials on the application of ECT in fragile populations are of the utmost importance, at a time where stigma toward its practice remains strong among health professionals and families (73, 74).

## Conclusion

After more than a century, catatonia remains a conundrum in the psychiatric field, with several definitions and classifications currently being used to describe a severe, less-rare-than-expected, clinical picture. P's case puts emphasis on the temporal variation of symptoms and their severity, not only in a recognizable pattern of “alternation” of extreme manifestations, such as stupor and psychomotor agitation, but in a more nuanced continuum, interrupted by periods of almost adequate functioning. We suggest that the presence of profound fluctuations of symptoms could be of great importance in the future definition, classification and treatment of this puzzling condition. Could catatonia be a chronic, although phasic, condition and could it be treated accordingly, with greater consideration toward duration and severity of symptoms?

## Data Availability Statement

The datasets presented in this article are not readily available because Data available on request due to privacy/ethical restrictions. Requests to access the datasets should be directed to annalisa.traverso@aopd.veneto.it.

## Ethics Statement

Written informed consent was obtained from the individual(s), and minor(s)' legal guardian/next of kin, for the publication of any potentially identifiable images or data included in this article.

## Author Contributions

AT wrote the manuscript, treated the patient, and collaborated in literature review. CA collaborated to the writing of the manuscript, literature research, and to treatment of the patient. SZ collaborated in discussion and in treating the patient. MG coordinated the research and clinical group. All authors contributed to the article and approved the submitted version.

## Conflict of Interest

The authors declare that the research was conducted in the absence of any commercial or financial relationships that could be construed as a potential conflict of interest.
